# Preparation and Characterization of Poly(δ-Valerolactone)/TiO_2_ Nanohybrid Material with Pores Interconnected for Potential Use in Tissue Engineering

**DOI:** 10.3390/ma12030528

**Published:** 2019-02-10

**Authors:** Waseem Sharaf Saeed, Abdel-Basit Al-Odayni, Ali Alrahlah, Abdulaziz Ali Alghamdi, Taieb Aouak

**Affiliations:** 1 Chemistry Department, College of Science, King Saud University, P.O. Box 2455, Riyadh 11451, Saudi Arabia; aalodayni@ksu.edu.sa (A.-B.A.-O.); aalghamdia@ksu.edu.sa (A.A.A.); 2 Restorative Dental Sciences Department, Engineer Abdullah Bugshan research chair for Dental and Oral Rehabilitation College of Dentistry, King Saud University, Riyadh 11545, Saudi Arabia; aalodayni@ksu.edu.sa

**Keywords:** poly(δ-valerolactone)/titanium oxide nanocomposite, cell adhesion, pore connection, Tissue Engineering, mechanical properties

## Abstract

Titanium dioxide/poly(δ-valerolactone) (TiO_2_/Pδ-VL) nanohybrid material containing interconnected pores with sizes in the range 80–150 μm were prepared by the solvent casting and polymer melting routes, and the dispersion of the TiO_2_ nanofiller in the Pδ-VL matrix and its adhesion were characterized by X-ray diffraction, differential scanning calorimetry, and scanning electron microscopy. A significant depression in the glass transition temperature (*T_g_*) and melting temperature (*T_m_*) values were revealed for the polymer nanocomposites prepared by the solvent casting technique. For the potential application of the prepared materials in the biomedical domain, complementary analyses were performed to examine the dynamic mechanical properties, and cell adhesion (using the 3-(4,5-dimethylthiazol-2-yl)-2,5-diphenyltetrazolium bromide (MTT) assay), and the results obtained for the samples prepared by the two methods were compared. Interconnected pores were successively produced in the new material by employing naphthalene microparticles as a porogen for the first time, and the results obtained were very promising.

## 1. Introduction

Recently, researchers in various fields have devoted much effort to the development of new polymeric materials capable of solving many problems in biomedicine, and particularly, in tissue engineering. Tissue engineering materials employed in the biomedical field are directly prepared from natural or synthetic polymers but for improvement in the biomedical properties, polymer blends or polymer composites have been devised.

Natural polymeric materials including polysaccharides, starch, alginate, cellulose, wool, silk, gelatin, and collagen are widely used in biomedical field because of their similar physicochemical performances in living systems similar to that of the native extracellular matrix [1]. These types of polymers are intensively used in the preparation of tissue engineering and drug carrier systems [2]. In living systems, these biopolymers demonstrate excellent biological performance, acceptable degradation rate, good biocompatibility, biological recognition, and tissue regeneration without scar or necrosis [3,4]. However, these materials also have disadvantages such as undesirable mechanical properties, likely transmission of pathogens, allergic reaction, limited availability, and high costs [5]. Recently, researchers have developed synthetic polymers to overcome the pitfalls associated with these materials. For instance, poly(glycolic acid) (PGA), poly(ε-caprolactone) (PCL), poly(lactic acid) (PLA), and poly(hydroxyl butyrate) (PHB) have been used either alone or in combination with other polymers or inorganic substances as scaffolds because of their desired properties, such as biodegradability, biocompatibility, and mechanical strength. However, these polymers lack cell recognition sites, and most of them have a hydrophobic character, preventing cell adhesion [6]. Among the aliphatic polyesters, poly(δ-valerolactone) (Pδ-VL), which is a member of the poly(lactone) family, has attracted less attention compared to PCL, although their physic-chemical and biomedical properties are practically similar. Pδ-VL has a semi-crystalline structure, low melting point (~−67 °C) and glass transition temperature fluctuates between 52 and 59 °C; in addition, it exhibits less elastomeric behavior compared to PCL [7]. Pδ-VL is slightly more hydrophilic than PCL, probably due to the presence of the polar carbonyl group, and fewer ethenyl groups (one group less) in the repetitive unit of Pδ-VL compared to that of PCL [8]. The degree of biodegradability of Pδ-VL (intermediate polyester) is in between that of poly(lactic-co-glycolic acid) (PLGA) and PCL. Pδ-VL, which has five methylene groups per monomeric unit, is usually prepared through the ring-opening polymerization route of δ-VL using different catalytic systems [9,10,11,12,13]. However, due to the toxicity of certain organometallic catalysts such as aluminum alkalates, tin carboxylates and certain complexes of group (III) are not tolerated in medical applications. Nevertheless, because of the good biodegradability, biocompatibility, and permeability characteristics of Pδ-VL, it is generally used as a block or hydrophobic sequence in amphiphilic copolymers recommended for the construction of micellar delivery systems of antitumor drugs with hydrophobic character [14]. 

To prevent the toxicity of these catalytic systems, researchers have extensively developed a new route based on enzymatic catalysis [15,16,17,18,19,20]. Despite the advantages of Pδ-VL, only a few studies have been published on the individual use of Pδ-VL in the medical domain. Pδ-VL is notably used in combination with others polymers [14], as well as for the regeneration of soft tissues and the manufacture of various clinical implants [21,22,23]. Despite the intensive efforts of different researchers in the biomedical field, the problem of controlling infections related to the biomedical materials exists, particularly, in dentistry because of the presence of bacteria and other microorganisms, which form biofilms on the surface of the biomaterials. Accordingly, antimicrobial activity, among other properties, is a major reason for the application of nanomaterials in dentistry [24,25]. Therefore, the development of a new biomaterial with antibacterial properties is expected to resolve much of the problem. For example, titanium dioxide (TiO_2_) particles have recently attracted a lot of attention in the biomedical field [26,27,28,29,30]. In addition, the TiO_2_ powder is effective for the formation of apatite on the surface of the PCL/TiO_2_ composite in simulated body fluids (SBF), a prerequisite for bioactivity. Liu et al. [31] have confirmed that nano-TiO_2_ effectively improves cell adhesion and proliferation. Similarly, via in vitro and in vivo studies, Goto et al. [32] have observed that the TiO_2_-containing bone cement not only regulates the setting time but also enhances the osteo-conductivity. TiO_2_, also known as “titania”, is usually obtained from a variety of ores. It is principally employed in pigments, adsorbents, catalyst supports, filters, coatings, photoconductors, and dielectric materials. The size of TiO_2_ particles is considered a key factor affecting its performance, particularly, when TiO_2_ is mixed with a polymer to obtain a composite material. Accordingly, several researchers have focused on the reduction of the size of TiO_2_ particles using different methods [33,34,35,36,37,38,39]. Nanostructured polymeric materials are considered promising materials for bone tissue engineering applications as they can simulate the nanometer surface roughness of natural osseous tissues [40,41,42,43,44,45]. Different investigations have revealed that the nanomaterials used in tissue engineering can be controlled at the molecular level, thereby enhancing the cell–material surface interactions [43]. Various studies have proven the advantages of nanoparticles of ceramics such as alumina, TiO_2_, and hydroxyapatite over the conventional micrometric ceramic particles with regard to the cellular behavior [39,46]. Superior cellular functions, particularly, osteoblast adhesion, improved alkaline phosphatase (biochemical marker for bone metabolism) synthesis, and enhancement in the calcium concentration (outside the cellular matrix) have been achieved with ceramic nanoparticles in a polymer matrix. For example, the PLGA/TiO_2_ nanocomposite is characterized with higher cytocompatibility than its homologue fabricated via the incorporation of TiO_2_ microparticles, i.e., the adhesion of chondrocytes and osteoblasts is found to be higher after incorporation of the nanoparticle filler in the polymer matrix [45]. These findings indicate that TiO_2_ nanoparticles can act as a substitute for the bioceramic microparticles usually employed as fillers in bioresorbable polymer scaffolds, such as bioactive glass or hydroxyapatite particles [47,48,49].

In this work, the nanocomposite Pδ-VL/TiO_2_ has been selected to acquire in-depth knowledge of the nature and physicochemical properties of this category of composite for its potential use in the biomedical field, and in particular in its applications in the field of tissue engineering in dentistry. Accordingly, Pδ-VL/TiO_2_ composites with variable loads of TiO_2_ nanoparticles were prepared by the solvent casting (SC) and polymer melting (PM) methods, and characterized by X-ray diffraction (XRD), differential scanning calorimetry (DSC), scanning electronic microscopy (SEM); in addition, the mechanical properties of the composites were examined by the dynamic mechanical analysis and tensile tests. The cell adhesion and growth to the surface of the prepared material was measured using the 3-[4,5-dimethylthiazole-2-yl]-2,5-diphenyltetrazolium bromide (MTT) assay.

## 2. Materials and Methods 

### 2.1. Chemicals

δ-VL (purity ~99.9%), nanopowder TiO_2_ (purity 99.7% and primary particle size 21 nm) were provided by Sigma Aldrich (Taufkirchen, Germany). Solvents and precipitants such as chloroform (purity ~99.9%), hexane (purity 99.5%) and isopropanol (purity 99.5%) were all supplied from BDH Prolabo. LoVo cells and Dulbecco’s modified Eagle’s medium (modified Eagle’s medium; DMEM), and fetal calf serum (FBS) were also provided by ATCC company (Manassas, VA, USA). Phosphate-buffered saline (PBS) and naphthalene beads used as a porogen agent were purchased from WANLAB company (Middlesex, UK). All chemicals were used without prior purification.

### 2.2. Synthesis of Pδ-VL)

10 mL (9.64 × 10^−2^ mol) of δ-VL was polymerized at 40 °C by ring opening route in the presence of 0.5 mL of HCl under a stream of nitrogen gas. A highly viscous solution was obtained at the end of the reaction, indicating the formation of Pδ-VL. The reaction was then stopped by pouring this solution dropwise into an excess of hexane, after which white Pδ-VL beads were obtained. The polymer thus obtained was purified by dissolution in tetrahydrofuran (THF) and precipitation several times in hexane. Subsequently, the as-obtained Pδ-VL was kept at 30 °C in a vacuum oven for complete drying, until a constant mass was reached. The average molecular weight of this polymer and its polydispersity index were measured by size exclusion chromatography in THF at 30 °C using a Varian apparatus (Palo Alto, CA, USA) equipped with a JASCO type 880-PU HPLC pump, UV and refractive index detectors, and TSK gel columns. This apparatus was pre-calibrated with a series of polystyrene standards, and the average number molecular weight and polydispersity index were determined to be 3.7 × 10^4^ g·mol^−1^ and 3.22, respectively.

### 2.3. Preparation of Pδ-VL/TiO_2_ Nanocomposite by SC Route

Pδ-VL (0.5 g) was dissolved in THF at 80 °C under continuous stirring until complete dissolution of the polymer. A known amount of TiO_2_ nanoparticles was added to the prepared solution, dispersed under stirring for 3 h, and then sonicated for 20 min to prevent agglomeration of the nanoparticles. The final suspension of Pδ-VL/TiO_2_ was then cast in a dish-shaped made of Teflon and the air bubbles were removed by stirring under low reduced pressure for 5 min. Then, the dish containing the degassed suspension was dried 25 °C for 24 h, then heated at a temperature of 50 °C in a vacuum oven for one day to completely remove traces of solvent. A series of Pδ-VL/TiO_2_ nanocomposites containing 1, 2, 3, 4, and 5 wt % TiO_2_ were prepared by the same procedure under the conditions illustrated in Table 1. 

### 2.4. Preparation of Pδ-VL/TiO_2_ Nanocomposite by PM Route

A certain amount of Pδ-VL was placed in a two-necked flask and heated at 63 °C under magnetic stirring until it was completely melted. A known amount of TiO_2_ nanoparticles was then added to the polymer in its liquid state. To avoid any possible degradation of the polymer, the flask was maintained in a nitrogen atmosphere until the end of the process. The mixture in the flask was homogenized by stirring for 20 min and then transferred to an ultrasonic bath. Subsequently, the resulting suspension was transferred to ambient temperature for solidification and nanocomposite formation. A series of Pδ-VL/TiO_2_ nanocomposites with different TiO_2_ contents were prepared by the same route and the preparation conditions are listed in Table 1.

### 2.5. Creation of Interconnected Pores

**Scheme 1:**
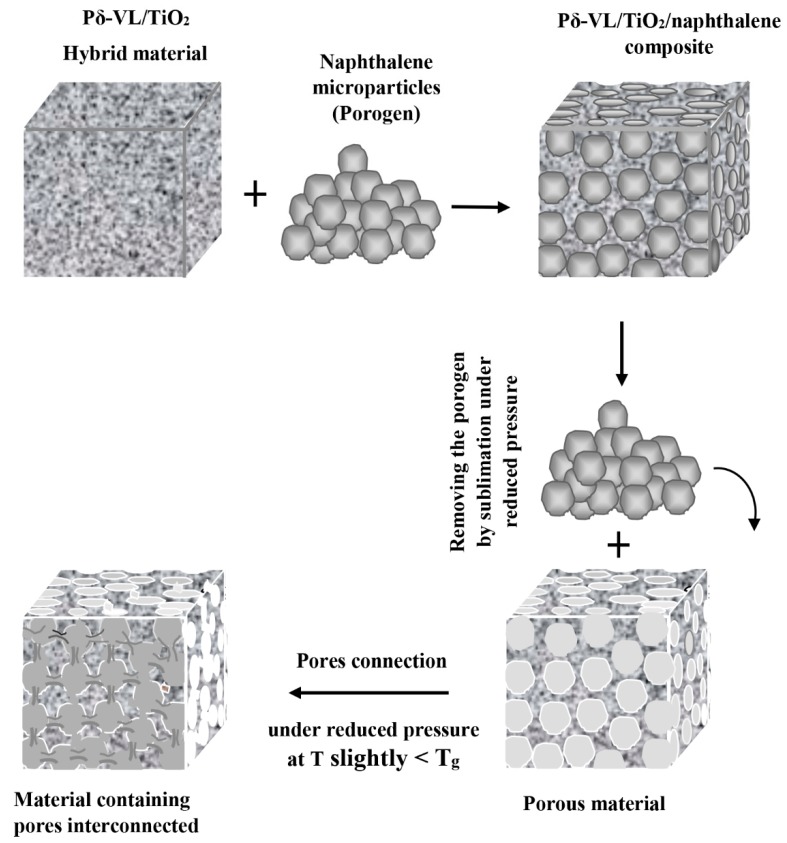
Fabrication of material containing pores interconnected using a sublimable porogen.

A certain amount of pure Pδ-VL or Pδ-VL/TiO_2_ hybrid material prepared by the SC method was dissolved in a minimum amount of THF at 30 °C under continuous stirring until complete dissolution. A quantity of naphthalene microparticles (150–250 μm) representing 60% by weight of the total amount was added to the mixture to obtain a highly viscous suspension. To ensure better dispersion of the TiO_2_ and naphthalene fillers in the polymer matrix, the assembly was placed in an ultrasonic bath for about 10 min at 30 °C. The bottom flask containing a pasty mixture was removed from the bath, and then, dried overnight at ambient temperature. To completely remove the residual solvent and porogen from the mixture and form microporous Pδ-VL/TiO_2_ hybrid material, the mixture was transferred to a vacuum oven maintained at 40 °C until a constant mass was attained. Notably, in these conditions, the naphthalene microparticles passed through their vapor state by sublimation. Interconnected pores were formed immediately after this step under vacuum at a temperature slightly below the glass transition temperature. Under these conditions, the walls separating the created pores became soft and easily disappeared under the effect of the high pressure difference inside and outside the pores, according to the process shown in Scheme 1. Note that, in order to completely remove all traces of incrusted naphthalene in the obtained porous interconnected material, the sample was then washed three times with hexane by immersion and with stirring in an excess of this solvent for 20 min, and then dried under vacuum for 24 h at 40 °C. The absence of any traces of naphthalene in the prepared material was proven by the total disappearance of the characteristic absorption bands in the UV–VIS and FTIR spectra. 

### 2.6. Characterization

#### 2.6.1. FTIR Analysis

The FTIR spectra of pure naphthalene, Pδ-VL/TiO_2_ hybrid material containing naphthalene (porogen) and after removing naphthalene are recorded by a Nicolet 6700 FT-IR, Thermo Scientific (Waltham, MA, USA). The reflectance spectra of each sample are directly collected between 400 and 4000 cm^−1^ without any treatment. The pure naphthalene was used as powder and the composites as films prepared by solvent casting. 

#### 2.6.2. XRD Analysis

The uniform dispersion of TiO_2_ nanoparticles in the Pδ-VL matrix was revealed by the XRD results and supported by the DSC analysis results. The crystalline structures of all specimens were examined on an X-ray diffractometer (RigakuDmax 2000, The Woodlands, TX, USA) with a Cu anode tube at a tube voltage/current of 40 kV/40 mA and generator current of 100 mA. All samples were scanned in the two theta range 5°–60° at a scan rate of 1.0 degree·min^−1^. The crystallite size of the TiO_2_ powder was determined using the Scherrer relationship [50,51]:

$$D=\frac{K\times \lambda }{\beta \cos\theta }$$

where λ represents the wavelength of the CuKα radiation which is 1.54 A and K is the form factor which takes the value of 0.90. *θ* and *β* are the Bragg angle and the half height of the angle diffraction, respectively. 

#### 2.6.3. DSC Analysis

The thermal behavior of the virgin PVL and Pδ-VL/TiO_2_ systems prepared by the two methods was studied using a Shimadzu DSC 60A (Kyoto, Japan) after having been calibrated with indium. Samples weighing between 10 and 12 mg were packed in aluminum pans before being placed in the DSC cell. The samples were scanned from −100 to + 200 °C under a nitrogen gas with a heating rate of 20 °C·min^−1^, then held at 200 °C for about five minutes to destroy all nuclei that could act like crystal seeds. All the data were collected from the first scan run; notably, none of the thermograms revealed any signs of decomposition. The glass transition temperatures, *T_gs_*, of the polymer and composites were accurately deduced from the inflection points of the thermal curves as the midpoint of the variation of the heat capacity versus temperature. The melting temperature, *T_m_*, was taken as the temperature corresponding to the maximum of the endothermic peak.

#### 2.6.4. SEM Analysis

The micrographs of the surface morphologies of the virgin polymer and its nanocomposite were examined by SEM (JEOL JSM 6360, Tokyo, Japan) with a 2 kV and 3 kV acceleration voltages. To reduce the surface charge, the film samples were carefully coated with a thin layer of gold using a JEOL JFC-1600 thin auto fine coater running at 20 mA for 80 s before examination. 

#### 2.6.5. Porosity and Pore Size Distribution

The porosity of the scaffolding was evaluated with a picknometer using *n*-decane (non-solvent for the polymer) as fluid of displacement as described in the literature [52]. The pore size and pore volume distributions of the prepared materials were evaluated using a mercury intrusion porosimeter Quartachrome, Poremaster 60, FL (London, UK). 

#### 2.6.6. Cell Adhesion and Growth Test

The effect of virgin polymer and hybrid material prepared by the two routes on LoVo cells was examined by the MTT assay described in the literature [53,54]. Accordingly, in plates containing six wells, 3 × 10^5^ cells (LoVo cells) were seeded and then exposed for 24 h at different concentrations of pure Pδ-VL and Pδ-VL/TiO_2_ nanocomposites. The culture medium thus prepared was then replaced by another containing 5 mg·mL^−1^ of MTT in PBS at a level of 10 volume % of the total volume of the culture and then incubated for 3 h at 37 °C. The produced formazan was then dissolved in 1.0 mL of a solution containing 0.1% by volume of isopropanol/hydrochloric acid mixture. Aliquot of 200 μL of the supernatant was then placed in a 96-well plate and the absorbance was recorded on an xMark Microplate Spectrophotometer (Bio-Rad, Hercules, CA, USA) at a wavelength of 550 nm. All experiments were performed using different positive controls for adhesion such as TCP (Do at 550 nm was 0.24) and a collagen-treated surface as well as non-adhesive surfaces as a negative control.

#### 2.6.7. Mechanical Properties Analysis

The DMA of virgin polymer and hybrid material prepared by the CS route were performed on DMA 290 (TA Instruments, Newcastle, UK). The specimens were scanned from −100 °C to 60 °C at a frequency of 1 Hz with a heating rate of 4 °C·min^−1^ and the length of specimens between the clamps was 47 mm. All the specimens were initially cooled at −110 °C using liquid nitrogen. Tensile tests were performed on pure Pδ-VL and the Pδ-VL/TiO_2_ hybrid material at body temperature (37 °C using an INSTRON 5566 universal testing apparatus, Norwood, ON, Canadawith a crosshead rate of 10 mm·min^−1^). The injection-molded samples were employed for the tensile measurements and the length of the gauge was 50 mm. The Shore A and D hardness test were performed on virgin Pδ-VL and Pδ-VL/TiO_2_ hybrid material following the ISO 868 [55] using a commercial durometer CEAST (Pianezza, Italy). The thickness of all specimens averaged between 4.2 and 4.6 mm; and measured points localized at 6 mm from the edge and the distance between the two extremities was 6 mm. All specimens were conditioned for two days at 23 °C at 50% RH before being examined.

## 3. Results and Discussions

### 3.1. FTIR Analysis

**Figure 1:**
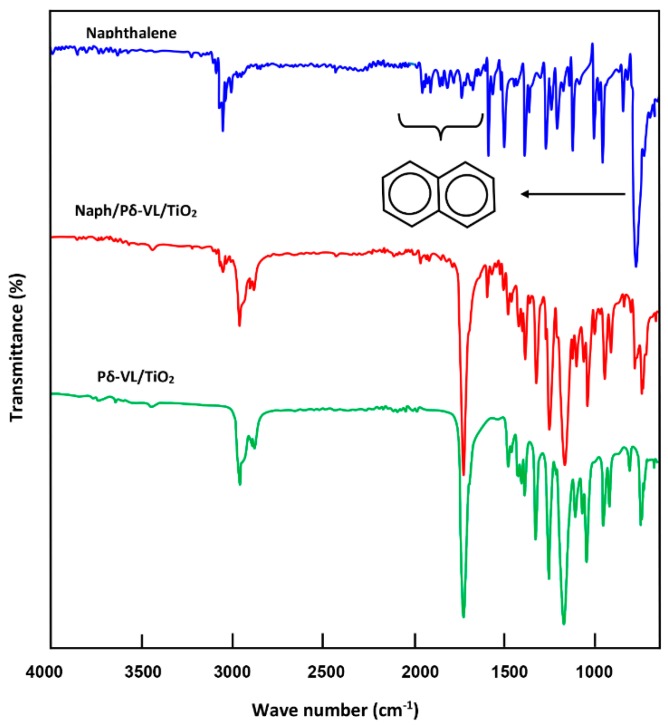
Comparative FTIR spectra of pure naphthalene, Pδ-VL/TiO_2_-1 hybrid material before and after removing the naphthalene.

The total disappearance of any traces of naphthalene incrusted in the interconnected porous of these materials was proved through the comparison of the FTIR spectra of the porous material with those of pure naphthalene and that of the materials containing the porogen prior to its extraction. For example, Figure 1 which shows the spectrum corresponding to PDVL /TiO_2_-1 system, after removing naphthalene, in which no signal is observed at 783 cm^−1^ characterizing its CH in-plane bending. 

### 3.2. XRD Analysis

**Figure 2:**
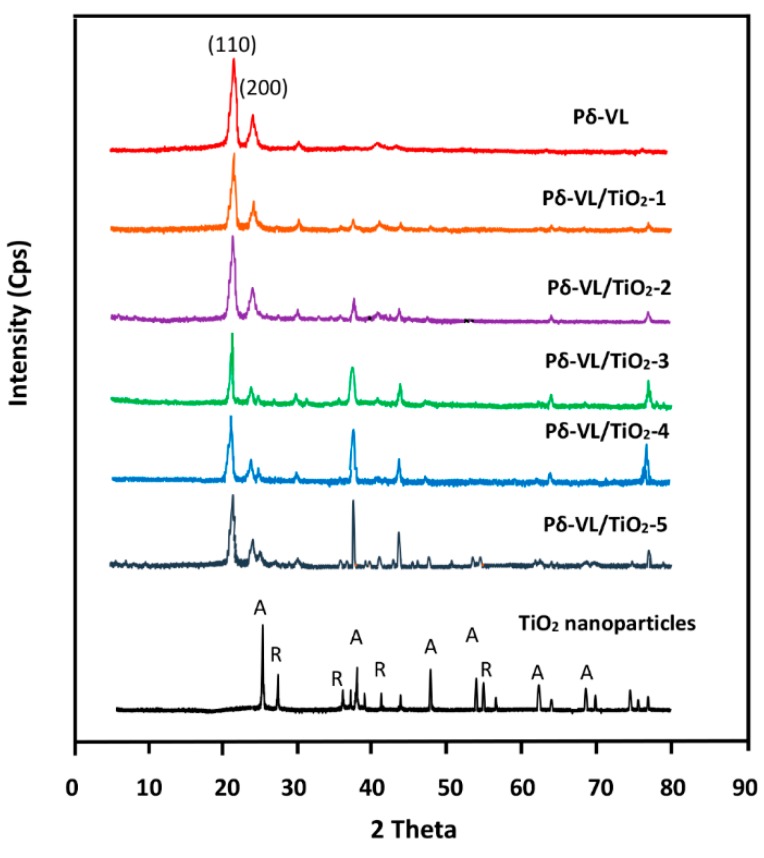
XRD patterns of pure TiO_2_ nanoparticles, virgin Pδ-VL and Pδ-VL/TiO_2_ nanocomposites with different TiO_2_ loads.

The XRD patterns of the prepared Pδ-VL/TiO_2_ hybrid materials and their pure components prepared by the SC and PM routes are identical (superposable), and one of them is shown in Figure 2. The spectrum of virgin Pδ-VL shows two crystalline peaks centered at 22° and 24° assigned to the diffraction of the (110) and (200) lattice planes, respectively [56], revealing an ordinary geometric crystalline structure. According to the results of the crystallographic analysis performed by Furuhashi on Pδ-VL, this polymer crystallizes in an orthorhombic cell structure [57]. The XRD pattern of TiO_2_ nanopowder reveals a mixture of the anatase and rutile phases, and the corresponding peaks marked in this figure agree with those of the standard spectrum of this compound [58]. The signal localized at 25.3°, representing the (101) characteristic peak of TiO_2_ anatase portion, was considered during the evaluation of the average crystal diameter; *β* was determined to be 0.411. The *β* values were converted to radians and the average crystalline size of the TiO_2_ powder determined using the Scherrer formula was 20 nm. However, the XRD traces of the Pδ-VL/TiO_2_ nanocomposite represents a combination of the patterns of the two pure components, thus revealing the non-formation of a new crystalline structure in the hybrid material. This finding proves the stability of the crystallinity of both the TiO_2_ nanoparticles and Pδ-VL in the nanocomposite. However, a significant deterioration in the crystallinity of Pδ-VL is revealed when the TiO_2_ content increased in the hybrid material. 

### 3.3. Thermal Behavior of Pδ-VL/TiO_2_ Nanocomposite

**Figure 3:**
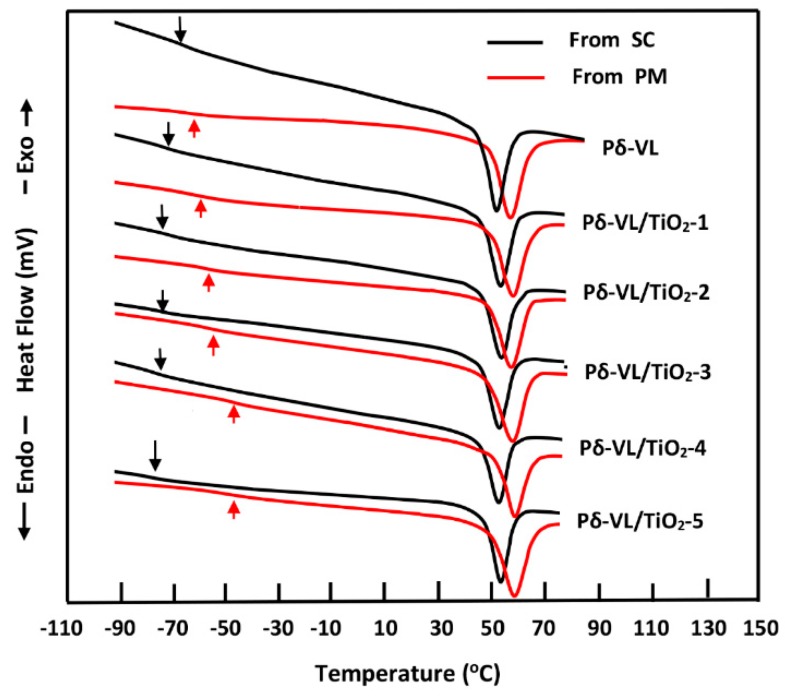
DSC thermograms of pure TiO_2_ nanoparticles, virgin Pδ-VL and Pδ-VL/TiO_2_ nanocomposites with different TiO_2_ loads prepared differently (SC and PM methods).

Figure 3 presents the comparison of the DSC thermograms of the Pδ-VL/TiO_2_ hybrid material prepared by solvent evaporation (SC) with those prepared by the PM process. As can be seen from these thermal traces, both methods revealed a significant shift in the *T_g_* and *T_m_* values of Pδ-VL in the hybrid material to low temperatures with the addition of the TiO_2_ nanocharge. Indeed, in the case of the hybrid material obtained by the SC route, the *T_g_* value of the Pδ-VL in the system varies from −75 °C to −68 °C with increase in the TiO_2_ load from 0% to 5% in the polymer matrix. On the other hand, *T_g_* varies from −63 °C to −47 °C for the material obtained by the PM route. In addition, the *T_g_* values of the nanocomposites obtained by the SC method are much lower than those of the samples prepared by the PM method. Nonetheless, in the melting zone, pseudo stability is observed for the *T_m_* value of Pδ-VL in the nanocomposite during the variation of the TiO_2_ nanocharge content, irrespective of the method used.

On the other hand, a comparison of the thermograms of the two series of hybrid materials prepared differently revealed a relatively large difference in the *T_m_* values; notably, the *T_m_* values of the samples obtained by the PM method are higher those of the samples obtained by the SC method. For example, *T_m_* of pure Pδ-VL prepared by SC is 52 °C, whereas, that of the sample obtained by PM is 56 °C. In addition, *T_m_* of the Pδ-VL/TiO_2_-3 sample with 3% by weight of TiO_2_ nanofiller prepared by SC is 52 °C, while that of the sample prepared by PM is 57 °C. The values of the thermal parameters *T_g_* and *T_m_* determined for virgin Pδ-VL obtained by the SC method perfectly agree with those reported in the literature [7,59]. The lower *T_g_* and *T_m_* values observed in the case of the SC-derived hybrid material compared to those of PM-derived hybrid material are probably due to faster sliding of macromolecular chains, which is attributed to the formation of nanopores left in the polymer matrix after evaporation of the solvent. In this case, the polymer chains will be more spaced from each other, facilitating their movement.

### 3.4. Assessment of Cell Adhesion and Growth

**Figure 4:**
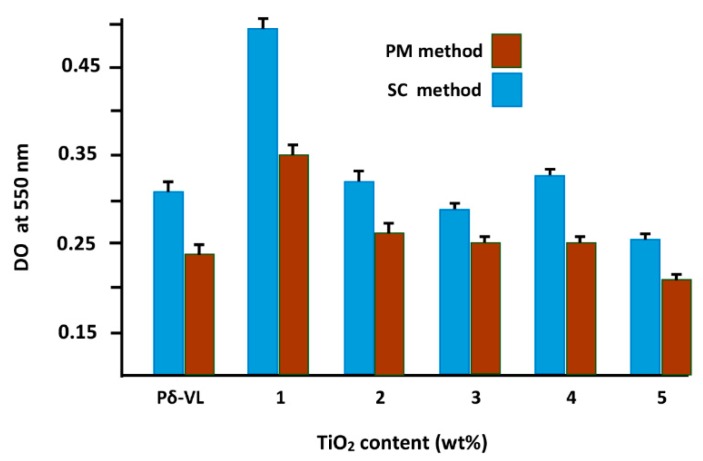
Cell adhesion screening tests performed with virgin Pδ-VL and TiO_2_/Pδ-VL/TiO_2_ hybrid materials with different TiO_2_ contents prepared by SC and PM routes.

**Scheme 2:**
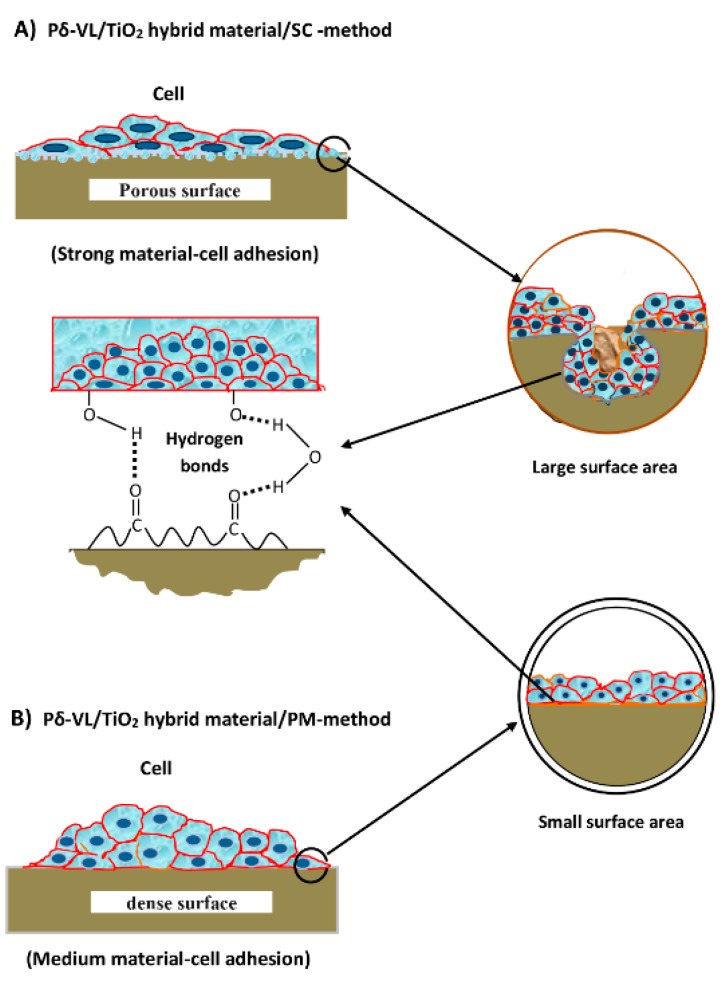
Comparative scheme of cell adhesion on Pδ-VL/TiO_2_ hybrid prepared differently: (**A**) SC-method (prepared by polymer melting); (**B**) PM-method (prepared by solvent casting).

The results of the evaluation of cell adhesion and growth performed on virgin Pδ-VL and Pδ-VL/TiO_2_ film samples with different compositions prepared by the SC and PM methods are shown in Figure 4. The results revealed that in the case of both the SC- and PM-derived samples, the LoVo-cell adhesion was more visible for the Pδ-VL/TiO_2_-1 nanocomposite, which contained 1 wt % of TiO_2_ content, after 24 h of culture compared to that for pure Pδ-VL and those containing a greater amount of the nanofiller. As shown in the figure, the comparison of the results of the cell adhesion tests on the two hybrid materials prepared differently indicates the best performance for the SC-derived samples. The enhanced cellular adhesion on the Pδ-VL/TiO_2_ specimen containing 1% by weight of TiO_2_ nanoparticles is probably due to an increase in the density of the pores on the surface of this sample (Scheme 2A) and an improvement in the wettability of Pδ-VL due to the formation of hydrogen bonds between the water molecules and the carbonyl groups of the Pδ-VL ester units on one side, and those between TiO_2_ and living cells on the other side (Scheme 2B). Comparable observation has been reported by Abdelwafa et al. [60] on PCL, and they have attributed this to the fact that the presence of a carbonyl ester or acid on the tissue engineering material promotes cell adhesion and proliferation in the protein-mediated cell adhesion mechanism. This finding additionally supports the proposal that the Pδ-VL/TiO_2_ hybrid material, especially, the nanocomposite containing 1% by weight of TiO_2_, is an effective potential candidate for tissue engineering applications in the biomedical field, for improved cell adhesion and cell growth.

To get an idea of the cell viability on this material, it is enough to compare the results obtained by Kiran et al. [61] on the hybrid material PCL/TiO_2_ nanocomposite. These authors report a viability of 82% with the material containing 2% by weight of TiO_2_ nanoparticles and decreased when this nanoinorganic filler increased to reach 53% with the material containing 7 wt % TiO_2_. Since Pδ-VL has a chemical structure comparable to that of PCL minus only one ethynil group(–CH_2_–), the results will also probably be equivalent.

### 3.5. Dynamic Mechanical Properties

**Figure 5:**
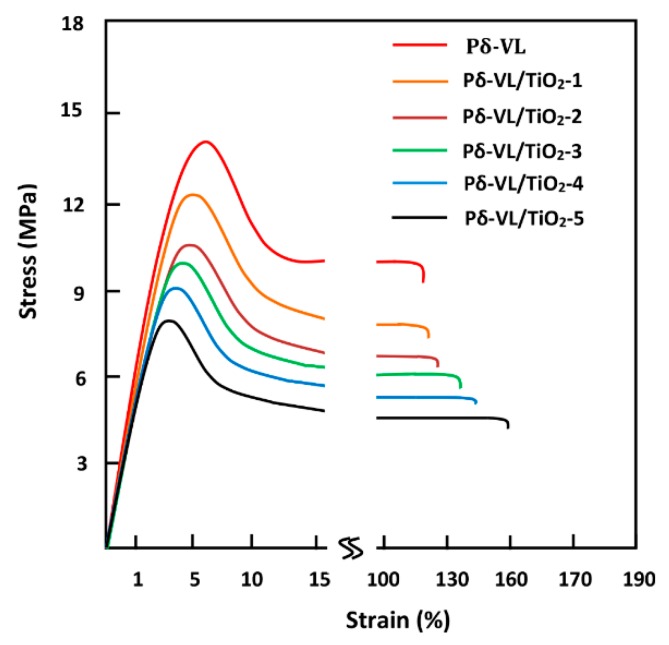
Typical stress–strain curve for pure Pδ-VL and Pδ-VL/TiO_2_ films, prepared by the SC method, undergoing tensile strain testing.

**Figure 6:**
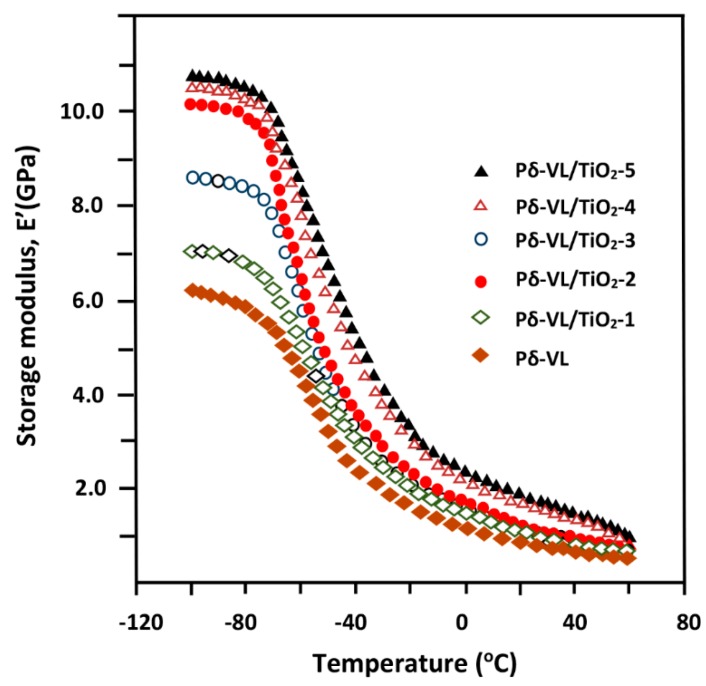
Variation of the storage modulus, *E*′, versus temperature for virgin Pδ-VL and Pδ-VL/TiO_2_ nanocomposites containing different TiO_2_ loads prepared by solvent casting method.

The dynamic mechanical properties of the virgin Pδ-VL polymer and Pδ-VL/TiO_2_ hybrid material containing different TiO_2_ loads prepared by the SC route are presented in Figure 5 and the data deduced from the curve profiles are listed in Table 2. A typical stress-strain curve is observed for pure Pδ-VL, and the yield point at 6.2% elongation and 14.2 MPa closely match with those reported by Aubin et al. [7] (i.e., 6% and 12.5 MPa, respectively). At larger elongations, the stress-strain curve shows necking and a plateau in the region between stress 10.2 MPa and the breaking point, which occurs at elongations between 118% and 160%. In addition, Young’s modulus of pure Pδ-VL is found to be 0.72 GPa. Aubin et al. have also reported that during the mechanical recovery experiments, Pδ-VL did not undergo permanent deformation at elongations of up to 2.2%. However, permanent deformations of 10 and 15% were observed for elongations of 4.0 and 5.0%, respectively. It is to be noted that a larger initial deformation leads to a significant permanent deformation. All Pδ-VL/TiO_2_ systems show a clearly recognizable yield point with increase in the TiO_2_ load; in addition, the tensile strength of the hybrid materials slightly shifts to higher values compared to that of virgin Pδ-VL. The effect of TiO_2_ nanoparticles on the viscoelastic properties of the Pδ-VL/TiO_2_ hybrid material was studied by conducting DMA measurements in the temperature ranged between −100 and 60 °C and the data obtained are shown in Figure 6. The storage modulus, *E*′, vs. the temperature curve of Pδ-VL revealed an enhancement in the dynamic mechanical properties (E′) of the material when the TiO_2_ nanofiller increased in the polymer matrix, indicating reinforcement effect.

As can be seen from these data, the Shore A and D values of the Pδ-VL/TiO_2_ nanocomposites are higher than that of pure Pδ-VL, which slowly increase with increase in the TiO_2_ content increased in the composites. This finding indicates a slight improvement in the Pδ-VL hardness after incorporation of nanoscale TiO_2_ into the polymer matrix.

### 3.6. SEM Analysis

**Figure 7:**
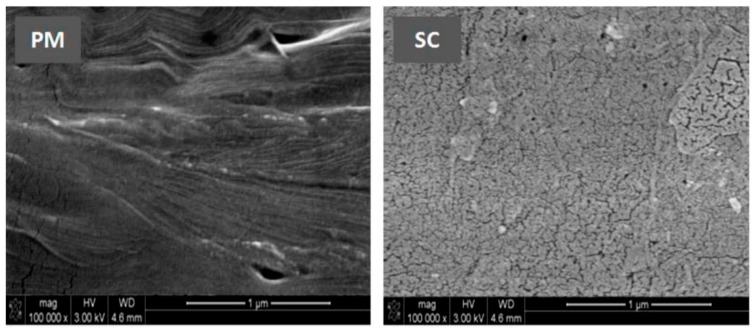
SEM micrographs of surface morphology of Pδ-VL/TiO_2_-1 hybrid material prepared differently: PM: prepared by polymer melting; SC: prepared by solvent casting.

Figure 7 shows the SEM micrographs of surface morphologies of Pδ-VL/TiO_2_-1 prepared by the CS (Image CS) and PM methods (Image PM). The comparison of the two images clearly shows two distinct surface morphologies. Indeed, the picture on the right, which corresponds to the hybrid material prepared by the SC method, shows a porous or furrowed surface resembling a lake left after evaporation of the water that contained it. On the other hand, this same system obtained by the MP method, on the left, exhibits a dense surface devoid of any traces of pores or furrows left after the fusion of Pδ-VL. This observation confirms the presence of high density of pores left after evaporation of the solvent when the material is prepared by the SC method. This observation supports our explanation of the adhesion of the cells on the material (Section 3.4). In fact, when the cells penetrate these cavities adhere to the inner wall of the material, due to the formation of hydrogen bonds created between the different substances, develop a very strong attachment to the material.

**Figure 8:**
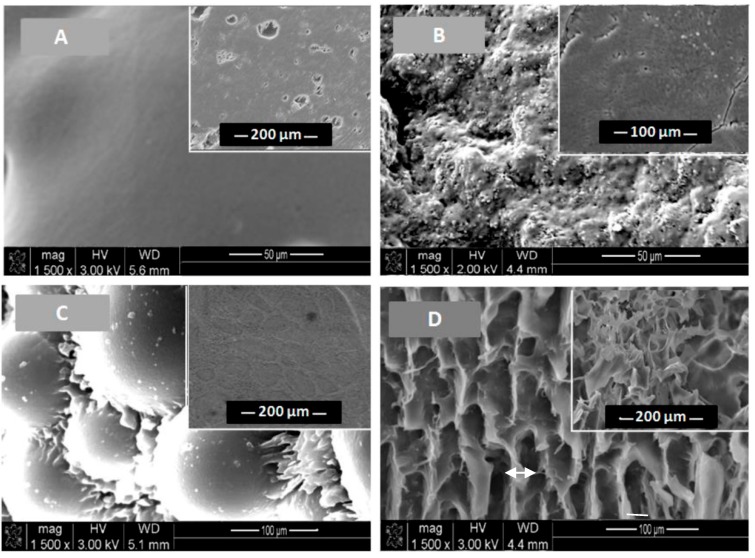
SEM micrographs of: (**A**) surface morphology of Pδ-VL film and its cross-sectional morphology(in right in the top); (**B**) surface morphology of Pδ-VL/TiO_2_-1 film and cross-sectional morphology (in the right in the top); (**C**) surface morphology of Pδ-VL/TiO_2_-1/ Naph and its cross-sectional morphology ( in the right in the top) before removing the porogen; and (**D**) surface morphology of Pδ-VL/TiO_2_-1/Naph film and its cross-sectional morphology (top right) of Pδ-VL/TiO_2_-1/ Naph film after removing the porogen then pores connection.

Figure 8 shows the SEM images of the surface and cross-section morphologies (top right) of the virgin Pδ-VL, Pδ-VL/TiO_2_-1, Pδ-VL/TiO_2_-1/naphthalene films prepared by the SC method before and after elimination of the porogen, which resulted in the formation of interconnected pores. As can be seen from Figure 8A, the pure Pδ-VL specimen shows a smooth surface devoid of any grafts or borrowings. The cross-sectional film (top right) also shows a similar morphology; however, some alveolus-sized (5–50 μm) structures non-uniformly dispersed in the polymer are observed inside the specimen. These microcavities are probably created by air bubbles trapped in the polymer during the sample preparation. The characteristics of multiphase systems such as nanocomposites are related to the nature and composition of the constituents, and influenced by the method of sample preparation. The micrographin Figure 8B presents the surface and cross-sectional morphologies of the Pδ-VL/TiO_2_-1 film, which was selected as the main sample in this study. This hybrid nanomaterial shows grainy surface morphology; the TiO_2_ nanoparticles appear dense, well covered with Pδ-VL, and uniformly dispersed in the polymeric matrix. This observation is well confirmed by the cross-section image of this sample (top right), showing TiO_2_ nanofiller particles uniformly dispersed in the Pδ-VL matrix. The adhesion between the particles and polymer seems to be quite high, as indicated by the absence of voids around the TiO_2_ filler. This finding confirms the good compatibility between TiO_2_ and the Pδ-VL polymer matrix, as revealed by the DSC analysis.

**Figure 9:**
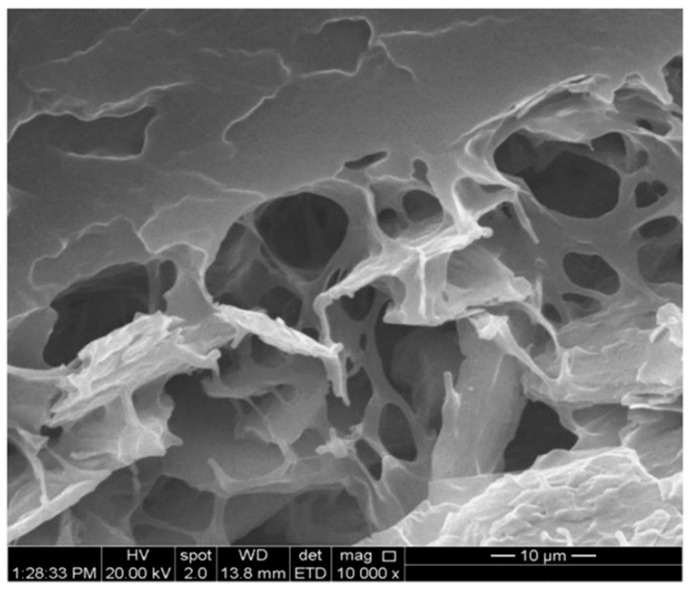
SEM micrograph of internal pores of PDVL/TiO_2_-1 hybrid material containing interconnected pores.

To understand the formation of interconnected pores within the Pδ-VL/TiO_2_-1 hybrid material, we compared the images taken before (Figure 8C) and after (Figure 8D) the removal of the porogen (naphthalene microparticles) from the nanocomposite material. Figure 8C presents the surface and the cross-section morphologies of the Pδ-VL/TiO_2_-1 films before the porogen removal. This micrograph shows a grainy surface morphology, wherein the porogen microparticles (80–150 μm) appear dense, well covered with the Pδ-VL /TiO_2_-1 nanocomposite, and uniformly dispersed in the polymeric matrix. The cross-section micrograph of this specimen (top right) revealed the same morphology observed on the surface, in which the porogen particles with the same size are seen dispersed. Figure 8D shows the surface and cross-sectional micrograph of Pδ-VL/TiO_2_-1 after removal of the porogen. This image shows a dense porous surface and cross section, in which most of the pores appear oval, with sizes between 30 × 100 μm^2^ and 60 × 120 μm^2^, and uniformly dispersed in the composite. The comparison between the size of the microporogen (150–250 µm) and that of the pores created by these microparticles revealed a size loss of about 20%. The narrowing of the pore size is probably caused by an excessive difference in the pressure inside and outside the pores during the pore connection. Figure 9 shows SEM image of the internal pores of this same specimen in which the pores interconnection is clearly presented. Indeed, as can be seen from this micrograph, the interconnection throats have a pseudo-circular or oval shape and their sizes vary between 4 and 15 μm.

### 3.7. Porosity and Pore Size Distribution

**Figure 10:**
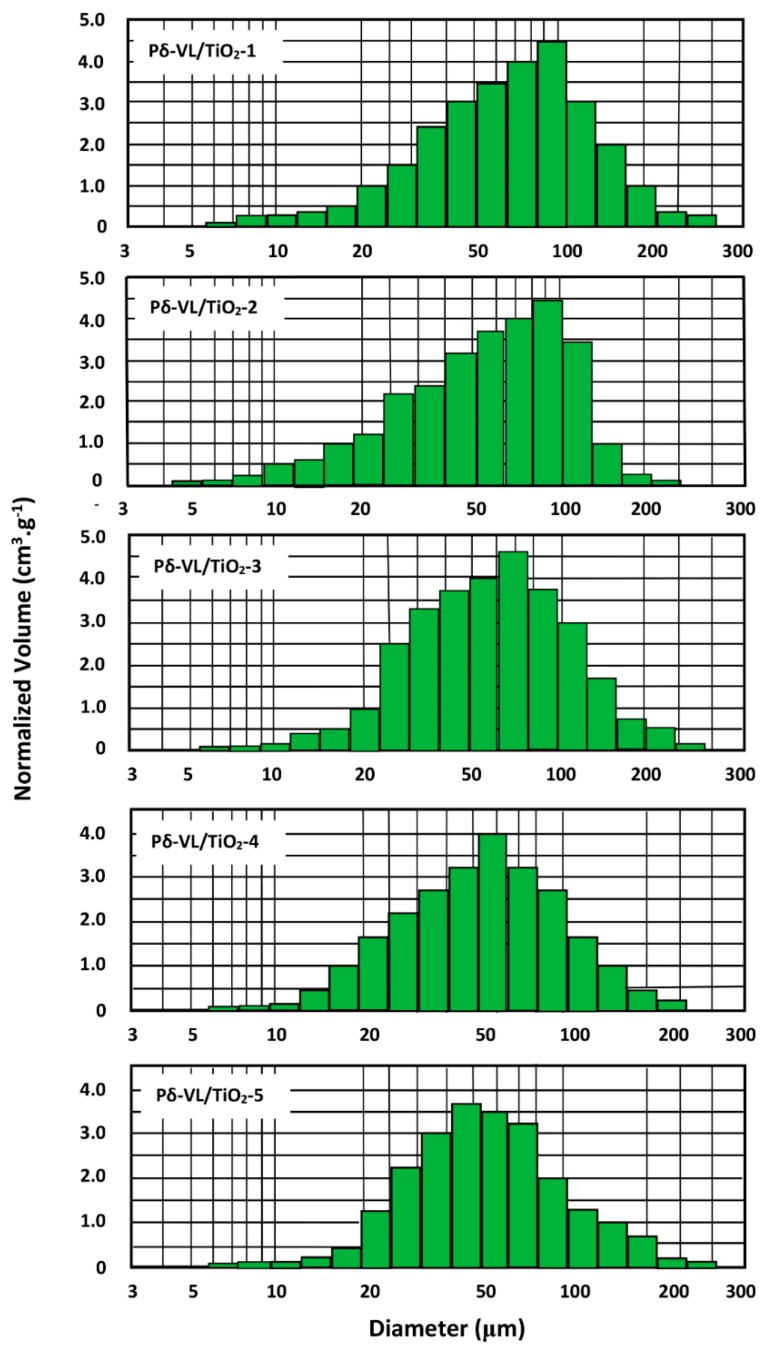
Pore sizes distributions in the microporous Pδ-VL/TiO_2_ hybrid materials with different TiO_2_ contents.

The structure of the scaffolds must be porous and the pores interconnected in order to facilitate the circulation of fluids transporting oxygen and nutrients from the cells. The porosity of the scaffolds determined by the pychnometer method are shown in Table 3. These values indicate an average porosity for the specimen containing 1 wt % of TiO_2_ samples with a value of 78.23%. For the sample containing 1 wt % of TiO_2_ and decreasing slightly with the increase of titanium oxide in the composite. This finding is probably related to the mechanical and thermal properties of the material. For example, the elongation at break and the *T_g_* values increased when the TiO_2_ content in the Pδ-VL/TiO_2_ material increased. In this sense the material becomes more and more flexible favoring a shrinkage of the pores due to the action of the high vacuum created during the formation of interconnected pores process. The data of the pore size distribution in the obtained Pδ-VL/TiO_2_ hybrid materials are illustrated in Figure 10. As can be seen from these values, all specimens showed a wide pore size distribution in which the top is localized between 30 and 100 μm depending on the TiO_2_ loaded in the composite. The presence of very small pores (<5 μm) can be a proof of the interconnection of the pores. In general, as shown in this figure the distribution of pores sizes is ranged between 4 and 270 μm. This result confirms clearly the presence of a high percentage of interconnected pores.

## 4. Conclusions

The main objectives of this investigation were practically achieved: a hybrid material based on Pδ-VL and TiO_2_ nanoparticles was successfully prepared by the SC and PM methods, and the use of naphthalene microparticles as a porogen was demonstrated for the first time. The combined effect of the physico-chemical and biological properties of the two components led to the production of a material with potential applications in the biomedical field. The characterization of the Pδ-VL/TiO_2_ hybrid material by XRD and DSC revealed stable crystallinity for the TiO_2_ nanoparticles in the material. A comparison of the thermal behavior of the polymer composites revealed significant depression in the *T_g_* and *T_m_* values for the sample obtained by the SC method. The *T_g_* value deduced from the DSC thermograms for Pδ-VL in the hybrid material significantly increased with increase in the TiO_2_ load. On the other hand, the *T_m_* value initially increased with increase in the TiO_2_ content, and then, stabilized at about 59 °C. Moreover, the comparative cell adhesion tests on the virgin polymer and hybrid materials revealed in general good viability and the maximum cell adhesion for the sample containing 1% by weight of the TiO_2_ nanofiller after 24 h of culture. This result confirmed the good compatibility between TiO_2_ nanoparticles and the Pδ-VL polymer matrix, as revealed by the DSC analysis. The dynamic mechanical properties of the Pδ-VL/TiO_2_ hybrid material showed a yield point at 6.2% elongation and 14.2 MPa. For larger elongations, necking was observed, and the stress-strain curve showed a plateau in the region between stress 10.2 MPa and the breaking point, which occurred at elongations between 118% and 160%. In addition, the tensile strength of the nanocomposites slightly shifted to higher values compared to that of the virgin Pδ-VL polymer. The effect of TiO_2_ nanoparticles on the viscoelastic properties of the Pδ-VL/TiO_2_ hybrid material studied by DMA in the range −100–60 °C indicating that the dynamic mechanical properties (*E′*) of Pδ-VL increased with increase in the TiO_2_ content in the Pδ-VL matrix, thereby revealing a reinforcement effect. The hardness parameters, Shore A and D values, of the TiO_2_/Pδ-VL hybrid material indicated a slight improvement in the Pδ-VL hardness after incorporation of nanoscale TiO_2_ in the polymer matrix. A comparative examination of the SEM images of Pδ-VL and Pδ-VL/TiO_2_-1 before and after the elimination of the porogen, which led to the formation of interconnected pores, revealed dense and uniform dispersion of TiO_2_ nanoparticles (well-covered and well-adhered) in the polymer matrix. A comparison of the sizes of the microporogen (150–250 µm) and that of the pores created by the microparticles revealed a loss in size of about 20%. 

All the results obtained in this study support the hypothesis that Pδ-VL/TiO_2_-1 hybrid material can be an effective candidate for tissue engineering applications in the biomedical field, for improved cell adhesion and cell growth, notably so when this material is prepared by the solvent casting method.

## Figures and Tables

**Table 1:** Preparation conditions of Pδ-VL/TiO_2_ nanocomposites by SC and PM methods.

System	TiO_2_ (g)	Pδ-VL (g)	TiO_2_ (wt %)
Pδ-VL/TiO_2_-1	0.005	0.495	1.0
Pδ-VL/TiO_2_-2	0.010	0.490	2.0
Pδ-VL/TiO_2_-3	0.015	0.485	3.0
Pδ-VL/TiO_2_-4	0.020	0.480	4.0
Pδ-VL/TiO_2_-5	0.025	0.475	5.0

**Table 2:** Mechanical properties of neat Pδ-VL and Pδ-VL/TiO_2_ systems.

Specimen	Yield Point (MPa)	Tensile Strength (MPa)	Elongation at Break (%)	Storage Modulus, E’ (GPa) at 37 °C	Shore A	Shore D
Pδ-VL	9.02	14.20	118	0.72	92.7	42.7
Pδ-VL/TiO_2_-1	8.31	12.30	120	0.91	93.6	43.2
Pδ-VL/TiO_2_-2	8.18	10.72	125	1.05	94.0	43.8
Pδ-VL/TiO_2_-3	8.32	10.04	137	1.12	94.5	44.9
Pδ-VL/TiO_2_-4	7.80	9.06	143	1.43	95.0	46.0
Pδ-VL/TiO_2_-5	6.73	8.12	160	1.63	95.1	46.2

**Table 3:** The porosity of Pδ-VL/TiO_2_ hybrid materials containing different TiO_2_ loads.

Sample	Porosity (%)
Pδ-VL/TiO_2_-1	78.2 ± 4.2
Pδ-VL/TiO_2_-2	75.3 ± 3.2
Pδ-VL/TiO_2_-3	74.8 ± 3.2
Pδ-VL/TiO_2_-4	74.2 ± 3.6
Pδ-VL/TiO_2_-4	73.8 ± 3.3

## References

[1] Mano J., Silva G., Azevedo H.S., Malafaya P., Sousa R., Silva S.S., Boesel L., Oliveira J.M., Santos T., Marques A. (2007). Natural origin biodegradable systems in tissue engineering and regenerative medicine: Present status and some moving trends. J. R. Soc. Interface.

[2] Aravamudhan A., Ramos D.M., Nada A.A., Kumbar S.G. (2014). Natural polymers: Polysaccharides and their derivatives for biomedical applications. Natural and Synthetic Biomedical Polymers.

[3] Okamoto M., John B. (2013). Synthetic biopolymer nanocomposites for tissue engineering scaffolds. Prog. Polym. Sci..

[4] McMahon R.E., Wang L., Skoracki R., Mathur A.B. (2013). Development of nanomaterials for bone repair and regeneration. J. Biomed. Mater. Res. B Appl. Biomater..

[5] Basha R.Y., Doble M. (2015). Design of biocomposite materials for bone tissue regeneration. Mater. Sci. Eng. C Mater. Biol. Appl..

[6] Armentano I., Dottori M., Fortunati E., Mattioli S., Kenny J. (2010). Biodegradable polymer matrix nanocomposites for tissue engineering: A review. Polym. Degrad. Stab..

[7] Aubin M., Prud’homme R.E. (1981). Preparation and properties of poly (valerolactone). Polymer.

[8] Gagliardi M., Di Michele F., Mazzolai B., Bifone A. (2015). Chemical synthesis of a biodegradable pegylated copolymer from ε-caprolactone and γ-valerolactone: Evaluation of reaction and functional properties. J. Polym. Res..

[9] Khalil M., Al-Shamary D., Al-Deyab S. (2015). Synthesis of poly (δ-valerolactone) by activated monomer polymerization, its characterization and potential medical application. Asian. J. Biochem. Pharm. Res.

[10] Vaida C., Takwa M., Martinelle M., Hult K., Keul H., Möller M. (2008). Γ-Acyloxy-ε-Caprolactones: Synthesis, Ring-Opening Polymerization vs. Rearrangement by Means of Chemical and Enzymatic Catalysis. Macromol. Symp..

[11] D’auria I., Mazzeo M., Pappalardo D., Lamberti M., Pellecchia C. (2011). Ring-opening polymerization of cyclic esters promoted by phosphido-diphosphine pincer group 3 complexes. J. Polym. Sci. A: Polym. Chem..

[12] Albertsson A.-C., Varma I.K. (2003). Recent developments in ring opening polymerization of lactones for biomedical applications. Biomacromolecules.

[13] Grobelny Z., Matlengiewicz M., Skrzeczyna K., Swinarew A., Golba S., Jurek-Suliga J., Michalak M., Swinarew B. (2015). Ring-opening polymerization of lactones initiated with metal hydroxide-activated macrocyclic ligands: Determination of mechanism and structure of polymers. Int. J. Polym. Anal. Charact..

[14] Nair L., Jagadeeshan S., Nair S.A., Kumar G.V. (2011). Evaluation of triblock copolymeric micelles of δ-valerolactone and poly (ethylene glycol) as a competent vector for doxorubicin delivery against cancer. J. Nanobiotechnol..

[15] Varma I.K., Albertsson A.-C., Rajkhowa R., Srivastava R.K. (2005). Enzyme catalyzed synthesis of polyesters. Prog. Polym. Sci..

[16] Kobayashi S., Makino A. (2009). Enzymatic polymer synthesis: An opportunity for green polymer chemistry. Chem. Rev..

[17] Kobayashi S. (2009). Recent developments in lipase-catalyzed synthesis of polyesters. Macromol. Rapid. Commun..

[18] Kobayashi S. (2010). Lipase-catalyzed polyester synthesis–A green polymer chemistry. Proc. Jpn. Acad. Ser. B..

[19] Kadokawa J.-I., Kobayashi S. (2010). Polymer synthesis by enzymatic catalysis. Curr. Opin. Chem. Biol..

[20] Yang Y., Yu Y., Zhang Y., Liu C., Shi W., Li Q. (2011). Lipase/esterase-catalyzed ring-opening polymerization: A green polyester synthesis technique. Proc. Biochem..

[21] Radhakrishnan J., Krishnan U.M., Sethuraman S. (2014). Hydrogel based injectable scaffolds for cardiac tissue regeneration. Biotechnol. Adv..

[22] Boucher H. (2017). Development and characterization of a polyester-based implant for controlled drug release. Master’s Thesis.

[23] Xu C., Huang Y., Tang L., Hong Y. (2017). Low-initial-modulus biodegradable polyurethane elastomers for soft tissue regeneration. ACS Appl. Mater. Interf..

[24] Sodagar A., Akhoundi M.S.A., Bahador A., Jalali Y.F., Behzadi Z., Elhaminejad F., Mirhashemi A.H. (2017). Effect of TiO_2_ nanoparticles incorporation on antibacterial properties and shear bond strength of dental composite used in Orthodontics. Dental. Press. J. Orthod..

[25] Sun J., Forster A.M., Johnson P.M., Eidelman N., Quinn G., Schumacher G., Zhang X., Wu W.-l. (2011). Improving performance of dental resins by adding titanium dioxide nanoparticles. Dent. Mater..

[26] Li Q., Wang X., Lu X., Tian H., Jiang H., Lv G., Guo D., Wu C., Chen B. (2009). The incorporation of daunorubicin in cancer cells through the use of titanium dioxide whiskers. Biomaterials.

[27] Paunesku T., Vogt S., Lai B., Maser J., Stojićević N., Thurn K.T., Osipo C., Liu H., Legnini D., Wang Z. (2007). Intracellular distribution of TiO_2_−DNA oligonucleotide nanoconjugates directed to nucleolus and mitochondria indicates sequence specificity. Nano Lett..

[28] Dimitrijevic N.M., Rozhkova E., Rajh T. (2009). Dynamics of localized charges in dopamine-modified TiO_2_ and their effect on the formation of reactive oxygen species. J. Am. Chem. Soc..

[29] Lu Z., Ye M., Li N., Zhong W., Yin Y. (2010). Self-assembled TiO_2_ nanocrystal clusters for selective enrichment of intact phosphorylated proteins. Angew. Chem. Int. Ed. Engl..

[30] Li J., Wang X., Jiang H., Lu X., Zhu Y., Chen B. (2011). New strategy of photodynamic treatment of TiO_2_ nanofibers combined with celastrol for HepG2 proliferation in vitro. Nanoscale.

[31] Liu H., Slamovich E.B., Webster T.J. (2005). Increased osteoblast functions on nanophase titania dispersed in poly-lactic-co-glycolic acid composites. Nanotechnology.

[32] Goto K., Tamura J., Shinzato S., Fujibayashi S., Hashimoto M., Kawashita M., Kokubo T., Nakamura T. (2005). Bioactive bone cements containing nano-sized titania particles for use as bone substitutes. Biomaterials.

[33] Sivakumar S., Pillai P.K., Mukundan P., Warrier K. (2002). Sol–gel synthesis of nanosized anatase from titanyl sulfate. Mater. Lett..

[34] Park S.D., Cho Y.H., Kim W.W., Kim S.-J. (1999). Understanding of homogeneous spontaneous precipitation for monodispersed TiO_2_ ultrafine powders with rutile phase around room temperature. J. Solid State Chem..

[35] Yin H., Wada Y., Kitamura T., Kambe S., Murasawa S., Mori H., Sakata T., Yanagida S. (2001). Hydrothermal synthesis of nanosized anatase and rutile TiO_2_ using amorphous phase TiO_2_. J. Mater. Chem..

[36] McCormick J.R., Zhao B., Rykov S.A., Wang H., Chen J.G. (2004). Thermal stability of flame-synthesized anatase TiO_2_ nanoparticles. J. Phys. Chem. B.

[37] Docters T., Chovelon J., Herrmann J., Deloume J. (2004). Syntheses of TiO_2_ photocatalysts by the molten salts method: Application to the photocatalytic degradation of prosulfuron. Appl. Catal. B Environ..

[38] Avvakumov G.V., Senna M., Kosova N.V. (2001). Soft Mechanochemical Synthesis: A Basis for New Chemical Technologies.

[39] Billik P., Plesch G. (2007). Mechanochemical synthesis of anatase and rutile nanopowders from TiOSO_4_. Mater. Lett..

[40] Savaiano J.K., Webster T.J. (2004). Altered responses of chondrocytes to nanophase PLGA/nanophase titania composites. Biomaterials.

[41] Kaplan F.S., Hayes W.C., Keaveny T.M., Boskey A., Einhorn T.A., Lannotti J.P., Simon S.R. (1994). Orthopaedic Basic Science.

[42] Webster T.J. (2003). Nanophase ceramics as improved bone tissue engineering materials. Am. Ceram. Soc. Bull..

[43] Webster T.J., Ergun C., Doremus R.H., Siegel R.W., Bizios R. (2000). Specific proteins mediate enhanced osteoblast adhesion on nanophase ceramics. J. Biomed. Mater. Res..

[44] Webster T.J., Ergun C., Doremus R.H., Siegel R.W., Bizios R. (2000). Enhanced functions of osteoblasts on nanophase ceramics. Biomaterials.

[45] Webster T.J., Siegel R.W., Bizios R. (1999). Osteoblast adhesion on nanophase ceramics. Biomaterials.

[46] Kay S., Thapa A., Haberstroh K.M., Webster T.J. (2002). Nanostructured polymer/nanophase ceramic composites enhance osteoblast and chondrocyte adhesion. Tissue Eng..

[47] Zhang R., Ma P.X. (1999). Poly (α-hydroxyl acids)/hydroxyapatite porous composites for bone-tissue engineering. I. Preparation and morphology. J. Biomed. Mater. Res..

[48] Boccaccini A.R., Maquet V. (2003). Bioresorbable and bioactive polymer/bioglass composites with tailored pore structure for tissue engineering applications. Compos. Sci. Technol..

[49] Blaker J., Gough J., Maquet V., Notingher I., Boccaccini A. (2003). In vitro evaluation of novel bioactive composites based on bioglass-filled polylactide foams for bone tissue engineering scaffolds. J. Biomed. Mater. Res. A.

[50] Scherrer P. (1912). Bestimmung der inneren Struktur und der Größe von Kolloidteil chenmittels Röntgenstrahlen, Kolloid Chemie EinLehrbuch.

[51] Patterson A. (1939). The scherrer formula for X-ray particle size determination. Phys. Rev..

[52] Endogan Tanir T., Hasirci V., Hasirci N. (2015). Preparation and characterization of Chitosan and PLGA-based scaffolds for tissue engineering applications. Polym. Compos..

[53] Semlali A., Jacques E., Rouabhia M., Milot J., Laviolette M., Chakir J. (2010). Regulation of epithelial cell proliferation by bronchial fibroblasts obtained from mild asthmatic subjects. Allergy.

[54] Semlali A., Chakir J., Goulet J.P., Chmielewski W., Rouabhia M. (2011). Whole cigarette smoke promotes human gingival epithelial cell apoptosis and inhibits cell repair processes. J. Periodont. Res..

[55] International Organization for Standardization (2003). ISO 868:2003, Plastics and Ebonite—Determination of Indentation Hardness by Means of a Durometer (Shore Hardness).

[56] Ren Y., Wei Z., Wu T., Bian Y., Leng X., Zhou C., Li Y. (2016). Synthesis of highly branched poly (δ-valerolactone)s: A comparative study between comb and linear analogues. RSC Adv..

[57] Furuhashi Y., Sikorski P., Atkins E., Iwata T., Doi Y. (2001). Structure and morphology of the aliphatic polyester poly (δ-valerolactone) in solution-grown, chain-folded lamellar crystals. J. Polym. Sci. Part B Polym. Phys..

[58] Thamaphat K., Limsuwan P., Ngotawornchai B. (2008). Phase characterization of TiO_2_ powder by XRD and TEM. Kasetsart J. Nat. Sci..

[59] Kasyapi N., Bhowmick A.K. (2014). Nanolamellar triblock of poly-d,l-lactide–δ-valerolactone–d,l-lactide with tuneable glass transition temperature and crystallinity for use as a drug-delivery vesicle. RSC Adv..

[60] Abedalwafa M., Wang F., Wang L., Li C. (2013). Biodegradable poly-epsilon-caprolactone (PCL) for tissue engineering applications: A review. Rev. Adv. Mater. Sci..

[61] Kiran A., Kumar T., Sanghavi R., Doble M., Ramakrishna S. (2018). Antibacterial and bioactive surface modifications of titanium implants by PCL/TiO_2_ nanocomposite coatings. Nanomaterials.

